# Construct Validity and Reliability of the OMNI Scale in Children and Adolescents With Neuromuscular Diseases

**DOI:** 10.1002/mus.70399

**Published:** 2026-09-09

**Authors:** Juliana Cardoso, Clara Corsete Tonoli, Beatriz Parra Buzzetti, Gabriela Barroso Davoli, Ana Claudia Mattiello‐Sverzut

**Affiliations:** ^1^ Department of Health Science, Ribeirao Preto Medical School University of Sao Paulo Sao Paulo Brazil

**Keywords:** Charcot–Marie–Tooth disease, Duchenne muscular dystrophy, myopathy, physical exertion, rehabilitation

## Abstract

**Introduction/Aims:**

Children and adolescents with neuromuscular diseases often demonstrate muscle weakness and mobility limitations, which may increase perceived exertion during functional tasks. The OMNI scale was developed to assess perceived exertion in pediatric populations; however, its measurement properties in neuromuscular conditions remain unclear. This study aimed to examine the construct validity of the OMNI scale, using the Borg scale as a reference, and its inter‐rater reliability during the 6‐minute walk test (6MWT) in children and adolescents with Charcot–Marie–Tooth disease (CMT) and myopathies.

**Methods:**

Construct validity was analyzed in 39 participants, and inter‐rater reliability in 21 participants aged 4–18 years. Perceived exertion was assessed before, during, and after the 6MWT using the OMNI and Borg scales. Linear mixed‐effects models examined construct validity. The intraclass correlation coefficient (ICC) and standard error of measurement (SEM) assessed reliability.

**Results:**

A significant association was observed between Borg and OMNI scores (*β* = 0.82, *p* < 0.001). OMNI scores increased throughout the 6MWT, indicating changes in perceived exertion. Inter‐rater reliability was moderate at the second minute (ICC = 0.67; 95% CI 0.34–0.85) and high at the end of the 6MWT (ICC = 0.75; 95% CI 0.48–0.89). The SEM was 1.74 points at the second minute and 1.32 points at the end of the 6MWT.

**Discussion:**

The OMNI scale demonstrated good construct validity and acceptable inter‐rater reliability, with low measurement error, supporting its clinical applicability. This suggests that the OMNI scale is a practical and reliable tool for monitoring perceived exertion in children and adolescents with neuromuscular diseases.

**Abbreviations**
6MWT: six‐minute walk test
CMT: Charcot–Marie–Tooth
CMTPedS‐Br: Brazilian Version of the Charcot–Marie–Tooth Pediatric Scale
HR: heart rate
ICC: intraclass correlation coefficient
MFM: Motor Function Measure

## 1 Introduction

Physical exertion can be measured using self‐perception scales, which, although subjective, are valid and standardized assessment tools. One of the most widely used instruments to measure self‐perceived physical exertion is the Borg scale, developed in 1982, which consists of verbal descriptors and a numerical scale [1].

Although it is used to assess different populations and contexts, such as monitoring physical exertion during the 6‐minute walk test (6MWT) [2] and evaluating asthma attacks [3], the structure of the scale does not appear to be appropriate for children, particularly younger ones, who often have difficulty interpreting verbal descriptors and assigning numerical values to sensations related to exercise [4].

These limitations led to the development of the OMNI scale. Composed of verbal descriptors, a numerical scale, and illustrations [5], it was specifically adapted to measure self‐perceived physical exertion in children. Since its creation, the OMNI scale has been validated for different populations and exercise modalities, such as cycling [5], treadmill walking [6], and resistance training [7]. Furthermore, its illustrations have been adapted and its descriptors translated into several languages, including Brazilian Portuguese [8, 9].

Despite its clinical relevance, there is still no validated version of the OMNI scale for neuromuscular diseases such as the Charcot–Marie–Tooth disease (CMT) and myopathies. In both conditions, clinical impairment leads to reduced levels of physical activities and quality of life [10, 11, 12].

Validating the OMNI scale for these neuromuscular diseases could meaningfully contribute to the assessment, prescription, and monitoring of physical rehabilitation, thus optimizing therapeutic approaches and improving functional outcomes. For a tool such as this to be effectively incorporated into clinical practice, it is essential to establish key measurement properties, including validity and reliability.

The aim of this study was to investigate the construct validity and the inter‐rater reliability of the OMNI scale in children and adolescents with CMT and myopathies. We hypothesized that the OMNI scale would demonstrate a positive association with the Borg scale and excellent reproducibility (Intraclass correlation coefficient [ICC] > 0.90).

## 2 Methods

### 2.1 Ethical Aspects

The Ethics Committee of the Hospital das Clínicas da Faculdade de Medicina de Ribeirão Preto da Universidade de São Paulo (HCFMRP‐USP) approved this study (Process number: 79018917.6.0000.5440 and 29925420.6.0000.5440). Informed consent was provided by study participants or their legal guardians.

### 2.2 Sample

This study used a convenience sample of children and adolescents with myopathies and CMT. Participants were recruited from the Physiotherapy Outpatient Clinic for Pediatric Myopathies and the Physiotherapy Outpatient Clinic for Pediatric CMT, both located at the Rehabilitation Center of the HCFMRP‐USP.

Participants aged 4–18 years, of both sexes, with a clinical diagnosis of any myopathy or type of CMT were included in the sample. Individuals with cognitive impairment, a history of upper or lower limb fractures within 1 year, associated cardiopulmonary diseases, and non‐ambulatory status were not included. Exclusion criteria were failure to understand the evaluator's commands or inability to complete the 6MWT.

### 2.3 Procedures

Assessments were conducted by trained evaluators at the Rehabilitation Center of the HCFMRP‐USP. Body mass index was obtained using bioelectrical impedance (Biodynamics 450, Seattle, USA), following the protocol described by Bell et al. [13]. For the CMT group, clinical impairment was assessed using the Charcot–Marie–Tooth Pediatric Scale, translated and validated into Brazilian Portuguese (CMTPedS‐Br) [14, 15]. This scale consists of 11 items, with total scores ranging from 0 to 44, where 0–14 indicate mild impairment, 15–29 moderate impairment, and 30–44 severe impairment. For the myopathies sample, the Motor Function Measure (MFM), also translated and validated into Brazilian Portuguese [16, 17], was used. This scale consists of 32 items, with a maximum total score of 96 points, where 0 indicates severe impairment and 96 indicates no impairment.

The level of physical exertion was measured using both OMNI and Borg scales before the 6MWT at rest, at the second minute of the test, at the end of the test, and in the third minute of recovery after the test. At each of these time points, the scales were presented to the participant sequentially by the evaluator in a randomized order.

The OMNI is a visual scale consisting of four illustrations showing a child or adolescent with progressively increasing effort [5]. Following the illustrations, in continuous order, are numerical ratings (0–10) and verbal descriptors (“not tired at all,” “a little tired,” “getting more tired,” “tired,” “really tired,” and “very, very tired”) [5, 9]. Validated OMNI verbal descriptors translated into Brazilian Portuguese were used in the present study [9]. During the assessment, children and adolescents indicated the illustration or descriptor that most closely resembled the intensity of their exertion.

The Borg scale [1] has already had its measurement properties tested in pediatric population [18]. It consists of continuous numerical ratings (0, 0.5, and 1–10) and verbal descriptors (“nothing at all,” “very, very weak,” “very weak,” “weak,” “moderate,” “somewhat strong,” “strong,” “very strong,” and “very, very strong”). The verbal descriptors were adapted from previous studies that translated the scale into Brazilian Portuguese [19]. During the assessment, children and adolescents indicated the number that best reflected the intensity of their exertion.

The 6MWT was performed following the protocol described by McDonald et al. [20]. All participants received standardized instructions on the procedures before starting the 6MWT. At any point during the test, the participant could stop to rest, as long as they remained standing and the timer continued to record time. If the participant experienced discomfort (chest pain, dyspnea, calf pain) or was unable to continue the test, even with encouragement from the evaluator, the test was interrupted and the timer reset. The total distance covered during the test was calculated using the following formula: (number of complete laps × 25) + distance covered in the last lap.

Heart rate (HR) was also measured using a digital oximeter (Geratherm oxy control, Geschwenda, Germany, GE Datex Ohmeda Tuffsat, Madison, USA) before the 6MWT, at the end of the test, and in the third minute of recovery after the test.

### 2.4 Inter‐Rater Reliability

Two independent evaluators examined the inter‐rater reliability of the OMNI scale. Each assessor had access only to the data obtained in their own assessment. Perceived exertion assessed by the OMNI scale was recorded during both the 6MWT test and retest. The sample size established for these analyses followed previous studies in neuromuscular diseases [15, 21]. The retest was administered following a minimum interval of 30 min and a maximum interval of 60 min.

### 2.5 Statistical Analysis

For statistical analysis, RStudio software version 2026.01.0+392 was used. The distribution of the sample data was explored using scatterplots. Evidence of construct validity was examined by analyzing the association between Borg and OMNI scores using linear mixed‐effects models fitted with the lme4 [22] package in R. The OMNI score was included as the dependent variable, Borg score as the main independent variable, and time (rest, second minute of the 6‐minute walk test, end of the test, and recovery) and diagnostic group (myopathy and CMT) as fixed effects. A random intercept for participants was included to account for repeated measurements within individuals. An interaction term between Borg score and diagnostic group was tested to assess whether the relationship between the two scales differed between groups. Models with and without the interaction term were compared using likelihood ratio tests. Model assumptions were evaluated through visual inspection of residual diagnostics, including linearity, homoscedasticity, normality of residuals and random effects, collinearity, and influential observations.

Inter‐rater reliability was assessed using the ICC, calculated based on a two‐way mixed‐effects model with absolute agreement for single measurements (ICC [1, 3]) [23]. The analysis was performed using the psych [24] package in R and considered values obtained at the second minute and end of the 6MWT. ICC values < 0.50 represent low reliability, 0.50–0.74 moderate, 0.75–0.90 high, and > 0.90 excellent [23]. Additionally, the standard error of measurement (SEM) and the minimal detectable change (MDC) were also quantified [25, 26]. The SEM [25] for both ICC estimates (second minute and end of the 6MWT) was calculated using the formula SD × √(1 − ICC), where SD represents the standard deviation of the OMNI score at the respective time points of the first assessment (test). The MDC [26] was calculated using the SEM derived from the ICC at the end of the 6MWT, applying the formula 1.96 × SEM × √2.

The correlations between OMNI and HR (at rest, end of the 6MWT, and recovery) and between OMNI and total distance of the 6MWT were calculated using Pearson's correlation coefficient (*r*). Values of *r* = 1.00 indicated perfect correlation; *r* = 0.90–0.80, strong; *r* = 0.70–0.50, moderate; *r* = 0.40–0.20, weak; and *r* = 0.10–0.00, no correlation [27].

## 3 Results

A total of 52 participants were included in the study (29 with myopathies and 23 with CMT). Eleven participants with myopathies and 2 with CMT were excluded because they did not understand the evaluator's commands or requested to end the walk test before the 6‐min mark. No relevant clinical complications were observed in the participants who did not complete the test. Therefore, the final sample consisted of 39 participants (18 with myopathies and 21 with CMT), of whom 20 (51.3%) were male.

### 3.1 Participants Characteristics

Table 1 presents the anthropometric characteristics and specific diagnoses of the study sample. According to the mean body mass index (BMI) values, the participants in the CMT group had a healthy weight, while participants in the Myopathies group were considered underweight.

**TABLE 1:** Anthropometric data and diagnostic information of study participants by analyzed group.

Anthropometric data	Total sample	Myopathies	CMT
	Mean (SD)	Mean (SD)	Mean (SD)
	(*N* = 39)	(*N* = 18)	(*N* = 21)
Age (years)	12.10 (3.7)	10.44 (2.6)	13.52 (3.9)
Body mass (kg)	46.11 (18.9)	38.27 (18.6)	53.54 (16.3)
Height (cm)	151.16 (17.8)	141.80 (14.2)	160.02 (16.6)
BMI (kg/m^2^)	19.22 (5.5)	18.18 (5.7)	20.31 (5.2)

Myopathy diagnosis	Number (%)
Duchenne muscular dystrophy (DMD)	5 (27.8)
Congenital muscular dystrophy	2 (11.1)
Becker muscular dystrophy	2 (11.1)
Mitochondrial myopathy	2 (11.1)
Limb‐girdle muscular dystrophy	2 (11.1)
Central core myopathy	1 (5.5)
Ullrich myopathy	1 (5.5)
Congenital myopathy	1 (5.5)
Glycogen storage disease myopathy	1 (5.5)
Unspecified myopathy	1 (5.5)

CMT diagnosis	Number (%)
CMT1A	14 (66.7)
CMT2A	2 (9.5)
CMTX1	2 (9.5)
CMT2C	1 (4.7)

Abbreviations: BMI, body mass index; CMT, Charcot–Marie–Tooth disease; SD, standard deviation.

On the MFM scale, the group with myopathies had a mean (standard deviation) of 86.5 (9.7) points. On the CMTPedS‐Br scale, the CMT group had a mean of 21.8 (6.0) points.

### 3.2 HR and Perceived Exertion Responses During the 6MWT

The HR values and Borg and OMNI scale scores obtained in the test and retest of the total sample, Myopathies and CMT groups before, during, and after the 6MWT are presented in Figure 1. The total distance covered at the end of the 6MWT is also presented in Figure 1.

**FIGURE 1:**
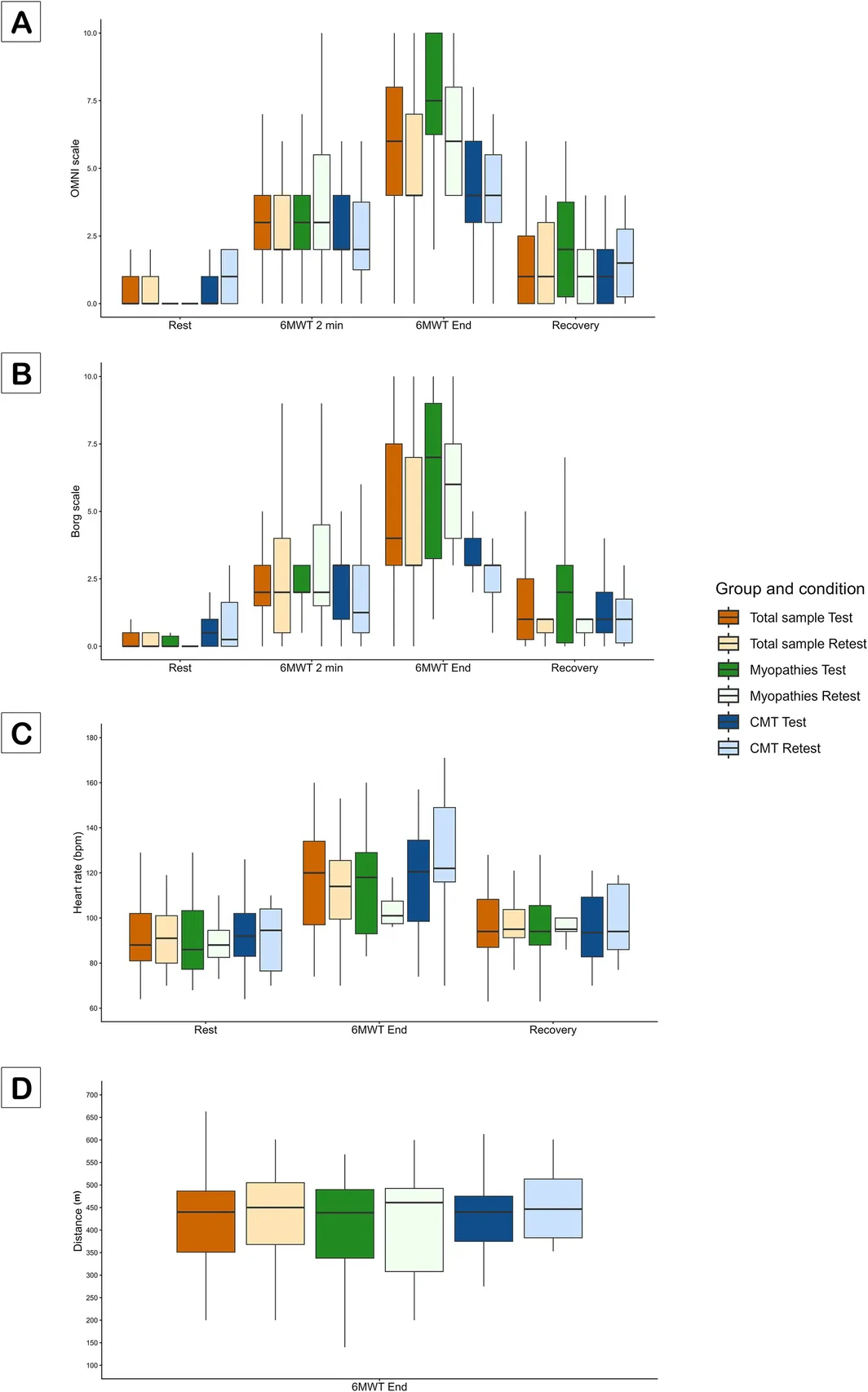
Measurements obtained during the 6MWT in the test and retest conditions. Based on descriptive analysis of the boxplot, both OMNI and Borg scales showed lower median values at rest, followed by a progressive increase at the second minute of the 6MWT and at the end of the test in both test and retest conditions. After the recovery period, both scales showed reduced median values. Heart rate followed a similar pattern across the rest, end of the 6MWT, and recovery periods. Total distance showed similar median values between groups.

Compared with rest, OMNI scores increased significantly at minute 2 and at the end of the test (Table 2). Regarding disease groups, no significant difference in OMNI scores was observed between participants with CMT and those with myopathy (Table 2).

**TABLE 2:** Factors associated with OMNI during the 6MWT.

Fixed effects	Estimate coefficient (*β*)	Standard error	*p*
Intercept	0.29	0.22	0.190
Borg score	0.82	0.04	< 0.001
Group (CMT vs. myopathy)	−0.28	0.28	0.330
Time—Minute 2 vs. rest	0.87	0.21	< 0.001
Time—End vs. rest	1.92	0.27	< 0.001
Time—Recovery vs. rest	0.23	0.20	0.230
Borg vs. group (interaction term)	−0.07	0.07	0.320

*Note:* OMNI score was considered as dependent variable and reference categories were rest for time and Myopathy for group.

Abbreviations: 6MWT, 6‐minute walk test; CMT, Charcot–Marie–Tooth disease.

### 3.3 Construct Validity

For construct validity the linear mixed‐effects model showed a strong and significant association between Borg and OMNI scores (*β* = 0.82, *p* < 0.001).

### 3.4 Correlations Between OMNI Scores, Heart Rate, and 6MWT Distance

Considering the total sample, no significant correlation was observed between OMNI and HR at rest (*r* = 0.2; *p* = 0.30).

### 3.5 Inter‐Rater Reliability

The anthropometric characteristics and specific diagnoses of this sample (*n* = 21/53%) are presented in Table S1. According to the reliability analysis, moderate reliability was observed at the second minute of the 6MWT (ICC = 0.67; 95% CI 0.340–0.850) and high reliability at the end of the test (ICC = 0.75; 95% CI 0.480–0.890). The SEM was 1.74 points at the second minute and 1.32 points at the end of the 6MWT. Based on the SEM derived from the ICC at the end of the 6MWT, the MDC was 3.67 points.

## 4 Discussion

A significant association between the OMNI and Borg scales was identified in this study, supporting the validity of the OMNI scale for assessing perceived physical exertion in children and adolescents with neuromuscular disorders. The inter‐rater reliability analysis demonstrated good reproducibility of the OMNI scale, partially confirming our initial hypothesis.

The OMNI scale is intended to quantify perceived exertion during physical activity [5], therefore we measured perceived exertion at different times during the 6MWT. Compared with rest, OMNI scores increased consistently at the second and sixth minutes of the test, indicating that the scale was able to detect changes in perceived physical exertion. The OMNI scale has previously been used to assess perceived exertion in typically developing children and adolescents during maximal treadmill exercise tests and has been shown to effectively reflect increases in exercise intensity [28, 29]. Our findings are consistent with this evidence and demonstrate that, in children and adolescents with neuromuscular diseases, although the 6MWT is considered a submaximal test [20], it was sufficiently demanding to elicit measurable changes in perceived exertion. Importantly, the purpose of using the 6MWT in this study was not to assess disease‐related fatigue, but rather to provide a standardized exercise stimulus capable of eliciting changes in perceived exertion.

In the present study, the OMNI scale measured the same construct of perceived exertion as the Borg scale. Specifically, for each one‐point increase in the Borg scale, the OMNI score increased by 0.82 points. The construct validity of the OMNI scale has previously been established through comparisons with the Borg scale [6, 30]. These studies demonstrated a positive association between OMNI and Borg ratings in adults during cycling and walking/running exercises [6, 30].

Regarding OMNI and HR, although both variables reflected changes in physical exertion during the 6MWT, only a non‐significant correlation was observed between them at rest. This finding differs from previous studies reporting strong correlations between OMNI and HR during incremental cardiopulmonary exercise tests in children and adolescents [5, 31, 32]. Unlike these studies, which used maximal or incremental protocols on cycle ergometers, the present study employed a submaximal, non‐incremental test. In line with our findings, Voorn et al. [33] reported that in adults with neuromuscular diseases, the relationship between HR and perceived physical exertion, measured using the Borg scale, differed when comparing maximal laboratory‐based exercise testing with aerobic home‐based training programs. These findings highlight that perceived physical exertion is a self‐reported measure, whereas HR represents a physiological response, and the relationship between them may vary depending on the testing context.

For reproducibility, the OMNI scale showed high inter‐rater reliability at the end of the 6MWT. A previous study reported excellent inter‐rater reliability of the OMNI scale in typically developing female adolescents during three treadmill exercise conditions [34]. Although we expected to observe similarly excellent reliability, some variability between test and retest may have been influenced by factors such as learning effects, motivation, and the subjective nature of perceived exertion, which may lead participants to report different scores across repeated assessments.

In addition, we observed a SEM of 1.32 points and an MDC of 3.67 points. These values indicate a relatively small measurement error and suggest that changes greater than approximately 3–4 points on the OMNI scale may represent a true change in perceived exertion. Although a difference of 3–4 points may appear large for a 0–10 scale, inspection of the boxplots in Figure 1A shows differences of a similar magnitude between different times of the 6MWT, supporting the clinical usefulness of the OMNI scale for detecting meaningful variations in perceived exertion.

It is important to acknowledge that the use of the OMNI scale requires adequate comprehension of the instructions and interpretation of perceived exertion ratings. This must be considered when using the scale. This study presents a few other limitations. Participants were not assessed under controlled environmental conditions (e.g., temperature, lighting, and humidity), and thus the potential influence of these factors on the results cannot be excluded. It was also not feasible to perform test–retest assessments on separate days due to the distance between participants' homes and the hospital where they received care. HR was not measured during the second minute of the 6MWT due to technical and operational limitations during data collection. A maximal cardiorespiratory exercise protocol was not employed, as individuals with neuromuscular disorders, particularly those with myopathies, often discontinue testing before reaching maximal effort. Accordingly, the 6MWT was selected, as it is better tolerated by individuals with CMT and myopathies. In addition, given the rarity of these conditions, the sample was small and heterogeneous. Despite these limitations, the findings support the use of the OMNI scale as a reliable and practical tool for assessing perceived exertion in children and adolescents with neuromuscular disorders as CMT and myopathies. Clinicians can apply the OMNI scale in practice to monitor exercise intensity, support exercise prescription, and improve patient engagement during functional tests such as the 6MWT.

## Author Contributions

**Juliana Cardoso:** conceptualization, methodology, data curation, investigation, formal analysis, visualization, writing – original draft, writing – review and editing. **Clara Corsete Tonoli:** conceptualization, investigation, methodology, data curation, visualization, writing – original draft. **Beatriz Parra Buzzetti:** conceptualization, methodology, investigation. **Gabriela Barroso Davoli:** conceptualization, methodology, investigation, writing – original draft. **Ana Claudia Mattiello‐Sverzut:** conceptualization, validation, methodology, project administration, resources, writing – review and editing, supervision.

## Funding

This work was supported by Fundação de Amparo à Pesquisa do Estado de São Paulo (2023/09029‐3, 2019/19878‐2, 2019/04369‐5, 2017/17596‐4); Coordenação de Aperfeiçoamento de Pessoal de Nível Superior (88887.837023/2023‐00).

## Ethics Statement

We confirm that we have read the Journal's position on issues involved in ethical publication and affirm that this report is consistent with those guidelines.

## Conflicts of Interest

The authors declare no conflicts of interest.

## Supporting information


**Table S1:** Anthropometric data and diagnostic information of participants in reliability analyses.

## Data Availability

The data that support the findings of this study are available from the corresponding author upon reasonable request.
