# Supplementary material for: Construct Validity and Reliability of the OMNI Scale in Children and Adolescents With Neuromuscular Diseases
Source: Muscle Nerve. 2026 Sep 9;74(5):1421–8. doi: 10.1002/mus.70399 (PMC13644902; doi:10.1002/mus.70399)
Supplement: Supplementary file 1 [file MUS-74-1421-s001.docx]

**Table.** Anthropometric data and diagnostic information of participants in reliability analyses

| **Anthropometric data** | **Total sample**  Mean (SD)  (N=21) | |
| --- | --- | --- |
| Age (years) | 13 (3.6) | |
| Body mass (kg) | 48.41 (20.2) | |
| Height (cm) | 153.44 (14.6) | |
| BMI | 19.85 (6.3) | |
| **Diagnosis** | | **Number (%)** |
| Duchenne muscular dystrophy (DMD) | | 2 (18.18%) |
| Congenital muscular dystrophy | | 2 (18.18%) |
| Becker muscular dystrophy | | 2 (18.18%) |
| Limb-girdle muscular dystrophy | | 2 (18.18%) |
| Central core myopathy | | 1 (9.09%) |
| Congenital myopathy | | 1 (9.09%) |
| Glycogen storage disease myopathy | | 1 (9.09%) |
| CMT1A | | 8 (80%) |
| CMTX1 | | 2 (20%) |
